# Endemic chikungunya fever in Kenyan children: a prospective cohort study

**DOI:** 10.1186/s12879-021-05875-5

**Published:** 2021-02-18

**Authors:** Doris K. Nyamwaya, Mark Otiende, Donwilliams O. Omuoyo, George Githinji, Henry K. Karanja, John N. Gitonga, Zaydah R. de Laurent, James R. Otieno, Rosemary Sang, Everlyn Kamau, Stanley Cheruiyot, Edward Otieno, Charles N. Agoti, Philip Bejon, Samuel M. Thumbi, George M. Warimwe

**Affiliations:** 1 KEMRI-Wellcome Trust Research Programme, P.O. Box 230-80108, Kilifi, Kenya; 2 KEMRI-Centre for Virus Research, Nairobi, Kenya; 3 Centre for Tropical Medicine and Global Health, University of Oxford, Old Road Campus, NDM Research Building, Oxford, OX3 7FZ UK; 4 Paul G Allen School for Global Animal Health, Washington State University, Pullman, WA 99164-7090 USA; 5 Centre for Global Health Research, Kenya Medical Research Institute, P.O. Box 1578-4100, Kisumu, Kenya; 6 Institute of Tropical and Infectious Diseases, University of Nairobi, P.O Box 19676, Nairobi, 00202 Kenya

## Abstract

**Background:**

Chikungunya fever (CHIKF) was first described in Tanzania in 1952. Several epidemics including East Africa have occurred, but there are no descriptions of longitudinal surveillance of endemic disease. Here, we estimate the incidence of CHIKF in coastal Kenya and describe the associated viral phylogeny.

**Methods:**

We monitored acute febrile illnesses among 3500 children visiting two primary healthcare facilities in coastal Kenya over a 5-year period (2014–2018). Episodes were linked to a demographic surveillance system and blood samples obtained. Cross-sectional sampling in a community survey of a different group of 435 asymptomatic children in the same study location was done in 2016. Reverse-transcriptase PCR was used for chikungunya virus (CHIKV) screening, and viral genomes sequenced for phylogenetic analyses.

**Results:**

We found CHIKF to be endemic in this setting, associated with 12.7% (95% CI 11.60, 13.80) of all febrile presentations to primary healthcare. The prevalence of CHIKV infections among asymptomatic children in the community survey was 0.7% (95% CI 0.22, 2.12). CHIKF incidence among children < 1 year of age was 1190 cases/100,000-person years and 63 cases/100,000-person years among children aged ≥10 years. Recurrent CHIKF episodes, associated with fever and viraemia, were observed among 19 of 170 children with multiple febrile episodes during the study period. All sequenced viral genomes mapped to the ECSA genotype albeit distinct from CHIKV strains associated with the 2004 East African epidemic.

**Conclusions:**

CHIKF may be a substantial public health burden in primary healthcare on the East African coast outside epidemic years, and recurrent infections are common.

**Supplementary Information:**

The online version contains supplementary material available at 10.1186/s12879-021-05875-5.

## Background

Chikungunya fever (CHIKF) is a mosquito-borne febrile illness characterised by acute, often chronic, debilitating polyarthralgia and polyarthritis that can last for months to years [1–3]. The disease is caused by chikungunya virus (CHIKV), a positive sense RNA virus in the family *Togaviridae* that was first isolated from a febrile patient in Tanzania in 1953 [4]. CHIKV transmission between humans is mainly mediated by the geographically widespread *Aedes aegypti* and *Ae. albopictus* mosquitoes, with geographic spread and spillover from sylvatic transmission cycles in monkeys thought to account for the periodic re-emergence of human disease outbreaks [5–8].

The CHIKV genome is approximately 11.8 kb in length and encodes four non-structural proteins (nsP1 to nsP4) required for viral replication, three major structural proteins (the capsid protein, and envelope proteins E1 and E2), and two small polypeptides (6 K/TF and E3) [9]. Exposure to CHIKV results in acquisition of protective virus neutralising antibodies that target the E2 protein, and is the basis for ongoing efforts to develop CHIKF vaccines [10, 11]. Phylogenetic studies have defined three CHIKV genotypes, namely East, Central and Southern Africa (ECSA), West Africa, and the Asian genotype [12]. In 2004, CHIKV re-emerged in coastal Kenya causing one of the largest epidemics on record, affecting millions of people as it spread along the Indian Ocean islands, India, southeast Asia and Europe [8, 13, 14]. This epidemic was associated with emergence of the Indian Ocean Lineage (IOL), which mapped within the ECSA genotype and included viruses with adaptive mutations in the E1 protein that increased their transmissibility by *Aedes albopictus* mosquitoes [15, 16].

CHIKF epidemics are now frequently reported globally [8, 17], but despite its original discovery and later re-emergence in East Africa, very little is known regarding inter-epidemic CHIKV exposure in the region. CHIKF cases have previously been detected in children in Kenya and Tanzania during inter-epidemic periods [18–20], which together with the high anti-CHIKV antibody seroprevalence observed in these settings suggest endemic CHIKV transmission [21, 22]. To address these knowledge gaps, we conducted a primary healthcare-based study linked to demographic surveillance and community survey to estimate the prevalence and incidence of CHIKV infections among children in Kilifi, coastal Kenya. Our study period, 2014 to 2018, included the most recent CHIKF epidemic year in Kenya, 2016 [23], allowing an unprecedented assessment of exposure risk and disease burden before, during and after a CHIKF epidemic in this setting.

## Methods

### Study setting and population

**Fig. 1:**
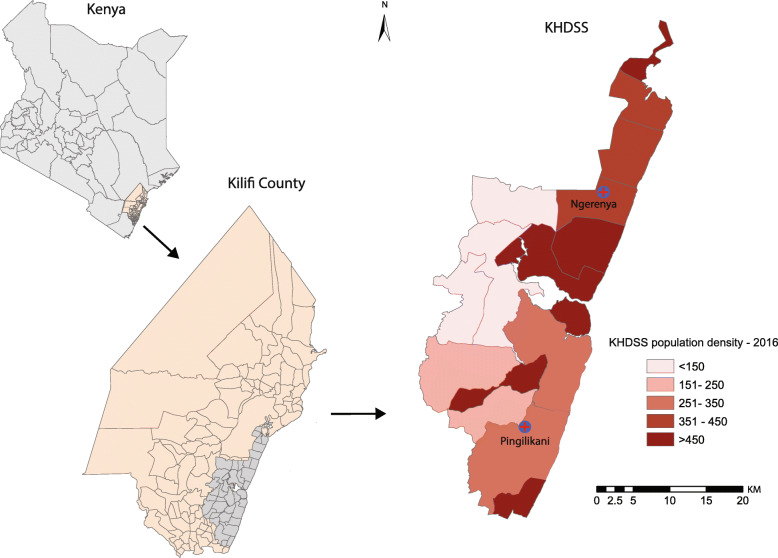
The Kilifi Health and Demographic Surveillance System. The location of Ngerenya and Pingilikani dispensaries within the KHDSS

We conducted the study between March 2014 and October 2018 in two dispensaries, Ngerenya and Pingilikani, both located in Kilifi County along the northern coast of Kenya (Fig. 1). The two dispensaries also lie within the Kilifi Health Demographic Surveillance System (KHDSS) boundaries which covers an area of 891km^2^ with approximately 290,000 residents that are enumerated every 4 months at the household level [24]. Linkage of clinical records at the dispensary and KHDSS surveillance data allowed estimation of disease incidence. The area under surveillance is endemic for malaria transmission and experiences two main rainy seasons; the ‘long rains’ during April to June and the short rains from October to December each year [25]. Residents living within the surveillance area are served by 21 public health facilities operating under the Kenya Ministry of Health guidelines. This includes Ngerenya and Pingilikani dispensaries located in the northern and southern parts of the surveillance area, respectively [24]. The catchment area of the two dispensaries have different rates of mosquito-borne pathogen exposure as inferred from previous malaria studies; Ngerenya and environs experiences low intensity malaria transmission, whereas malaria transmission around Pingilikani is moderate [26].

### Sample selection and processing

We drew a random sample of 3500 out of 5669 children, aged < 16 years, who visited the two dispensaries with fever (defined as axillary temperature of ≥37.5 °C) during the study period. Random sampling of individuals was done using the *runiform* function in STATA/IC version 15.1 (StataCorp College Station, Texas, USA). Finger-prick whole blood samples were collected in EDTA vacutainers from all febrile children for the purpose of malaria rapid diagnostic testing as part of an ongoing malaria surveillance study [27]. The remaining whole blood was stored at − 80 °C until the day of viral RNA isolation (see RT-PCR section below). No other samples were collected. Clinical diagnoses following investigations done at the dispensaries were provided by non-study clinicians.

We seperately conducted a community cross-sectional survey of 435 asymptomatic children aged < 16 years whose residence was within the catchment of the two dispensaries. Serum was obtained from these children and stored at − 80 °C until the day of viral RNA isolation. This cross-sectional community sampling was conducted between April and May 2016 and allowed estimation of the frequency of asymptomatic CHIKV infections in the study population. Ethical approval was provided by the Kenya Medical Research Institute Scientific and Ethics Review Unit and written informed consent obtained from parents or guardians of all study participants (SSC Nos. 3296, 2617 and 3149).

### CHIKV RT-PCR assays

CHIKV infection was defined as detection of CHIKV viral RNA by reverse-transcriptase polymerase chain reaction (RT-PCR) using a published primer-probe set targeting the CHIKV non-structural protein 1 (nsP1) region [28], namely CHIKV 874 (5′-AAAGGGCAAACTCAGCTTCAC-3′), CHIKV 961 (5′-GCCTGGGCTCATCGTTATTC-3′) and CHIKV 899-FAM (5′-*FAM*-CGCTGTGATACAGTGGTTTCGTGTG-*TAMRA*-3′). Briefly, 100 μl of finger-prick whole blood samples collected at presentation to the dispensaries was used for total RNA isolation using TRIzol™ Reagent (ThermoFisher) as per manufacturer instructions. Presence of CHIKV viral RNA was then determined using the QuantiFast RT-PCR Kit (Qiagen) in a 25 μl reaction with primers and probe at a final concentration of 100 nM and 20 nM, respectively, and 5 μl total RNA template. RT-PCR assays were done on a 7500 Real-Time PCR System (Applied Biosystems) with cycling conditions as follows: 50 °C for 20 min, 95 °C for 15 min, followed by 45 cycles of 94 °C for 15 s and 60 °C for 1 min. A second confirmatory screen was performed on some of the RT-PCR positive samples using an assay targeting the CHIKV nsP4 region as previously described [29]. For both assays, a positive result was defined as a cycle threshold (Ct) value of < 40. Viral RNA from a cultured CHIKV isolate obtained from a febrile Kenyan patient (GenBank accession: MT526796) was used as a positive control, while RT-PCR mastermix without template was used as a negative control.

### CHIKV genome sequencing and analysis

RT-PCR positive CHIKV samples were sequenced using the Nanopore MinION technology following PCR amplification using methods described in the PrimalSeq approach [30]. Full details of our sequencing and bioinformatics workflow can be found in the Supplementary Material. In brief, CHIKV-specific multiplex primers were designed using the Primal Scheme software and used for pre-enrichment of CHIKV viral RNA in the samples using a multiplex PCR method. Barcodes and sequencing adapters were ligated into each sample, after which all samples were pooled into a single reaction tube and the library loaded on to a MinION sequencing device for sequencing. The sequences generated in this study were deposited in GenBank, accession numbers: MT526798-MT526807. MUSCLE was used to align the Kilifi CHIKV genome sequences with those available in GenBank from other geographical locations (see Supplementary Material). Maximum Likelihood phylogenies were reconstructed from the alignment using RAxML [31] using the GTR substitution model with 4 gamma categories (GTR + G4) and visualized in FigTree v1.4.4.

Based on previous studies on childhood malaria [32], we expected that some children would present to the dispensaries with fever on multiple occasions during the study period. We therefore restricted our CHIKV RT-PCR screening to the earliest febrile episode (‘index episode’) for each of the 3500 children. However, to identify occurrence of recurrent infections, CHIKV RT-PCR screening was also done on all samples from subsequent febrile illnesses among children that were CHIKV positive at the index febrile episode. We defined recurrent CHIKV infection as occurrence of more than one episode of fever accompanied by a CHIKV RT-PCR positive test in the same individual.

### Statistical analyses

Data from the dispensaries were linked to the larger KHDSS data using unique person identifiers allowing us to estimate disease incidence in the population, and to assess the influence of various demographic variables on CHIKF incidence. We decided, a priori, to restrict the incidence analyses to a 5 km radius around each dispensary which we assumed to be the catchment area. Because we only sampled from all the visits (with febrile illness) made to the dispensaries, we estimated what the expected cases of CHIKF (out of all visits due to febrile illness) would be by multiplying the test positive rate from the sample by the total number of visits due to febrile illness. This was based on the assumption that the test positive rate among the randomly selected would hold true for the remaining unsampled visits due to febrile illness. Incidence rate was then computed as total number of expected CHIKF cases divided by total person years of observation (pyo) and was expressed per 100,000 pyo. Incidence rate ratios (IRRs) comparing incidence across various socio-demographic variables were computed using a univariate negative-binomial regression model. All statistical analyses were conducted using STATA/IC version 15.1 (StataCorp College Station, Texas, USA).

## Results

### CHIKV infections are common among children in coastal Kenya

**Fig. 2:**
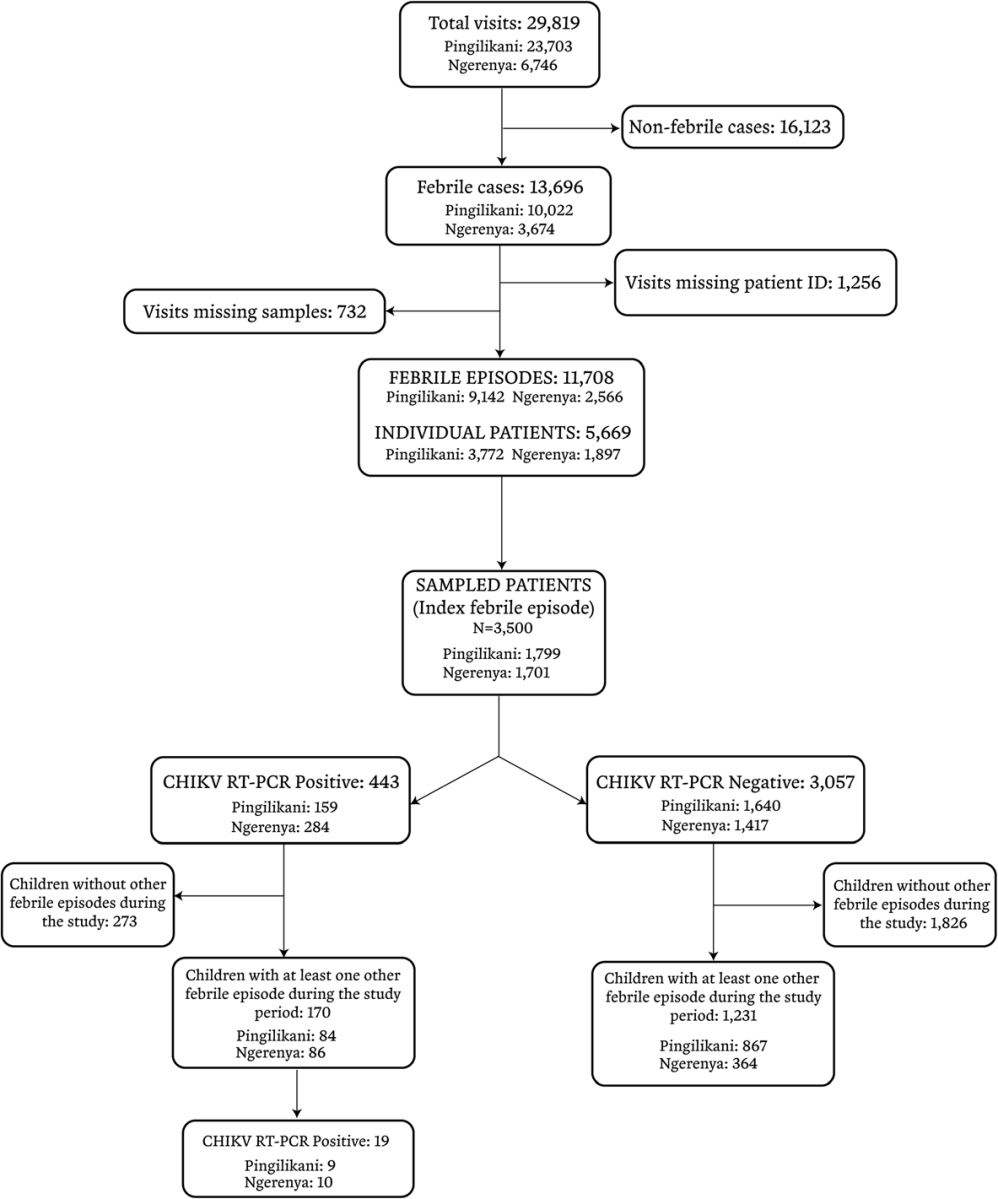
Study population. The flow of study participants, reasons for exclusion and results from CHIKV RT-PCR screening are shown

Between March 2014 and October 2018, there were 29,819 visits by children aged less than 16 years to the two dispensaries (6746 in Ngerenya, 23,073 in Pingilikani), of which 13,696 were febrile (Fig. 2). Of the 13,696 fevers, 1256 lacked a unique patient identifier and 732 visits were missing samples. After these exclusions, there were 11,708 febrile visits from a total of 5569 children (median 1 febrile visit per child during the study period, IQR 1–2) eligible for CHIKV RT-PCR analysis (Fig. 2).

The median age of the 3500 children was 3.1 years (IQR 1.3–6.4), with 1701 being resident in Ngerenya and 1799 in Pingilikani, respectively (Fig. 1). Of the 3500 children 443 were RT-PCR positive (12.7, 95% CI 11.60, 13.80; Table 1), with RT-PCR positivity being twice as frequent in Ngerenya (16.7, 95% CI 15.00, 18.54) than in Pingilikani (8.8, 95% CI 7.61, 10.24). CHIKF prevalence showed no statistically significant variation by sex or age, and in no instance was a clinical diagnosis of CHIKF assigned (Table 1). No clinical diagnosis was significantly overrepresented among CHIKF cases (Table 1). CHIKF prevalence was highest in 2016, when an epidemic was reported in Kenya [23], and was significantly lower in the pre- and post-epidemic years where it ranged between 2 to 6% in Ngerenya and 6 to 10% in Pingilikani (Table 1). CHIKF was more prevalent during January to March in Ngerenya (coinciding with the dry season; see Figure S1) but this was less apparent in Pingilikani (Table 1).

**Table 1:** Demographic characteristics of 3500 febrile children screened for CHIKV infection

	Ngerenya dispensary (***N*** = 1701)		Pingilikani dispensary (***N*** = 1799)	
	CHIKF cases n/N (%)	***P*** value (Chi2 test)	CHIKF cases n/N (%)	***P*** value (Chi2 test)
**Sex**		0.55		0.07
Female	132/763(17.3)		89/884 (10.1)	
Male	152/938 (16.2)		70/915 (7.6)	
**Age (years)**		0.27		0.67
< 1	54/260 (20.8)		25/341 (7.3)	
1 to < 5	151/926 (16.3)		69/774 (8.9)	
5 to < 10	66/430 (15.3)		43/469 (9.2)	
10 to 15	13/85 (15.3)		22/215 (10.2)	
**Year**		< 0.001		0.05
2014	No data		43/509 (8.4)	
2015	11/503 (2.2)		37/366 (10.1)	
2016	165/537 (30.7)		43/365 (11.8)	
2017	91/370 (24.6)		17/304 (5.6)	
2018	17/291 (5.8)		19/255 (7.4)	
**Season**		< 0.001		0.15
Jan – Mar	93/423(22.0)		49/432 (11.3)	
Apr – Jun	76/392 (19.4)		58/678 (8.5)	
Jul – Sep	66/496 (13.3)		31/443 (7.0)	
Oct – Dec	49/390 (12.6)		21/246 (8.5)	
**Clinical diagnosis**		0.58		0.06
Other^a^	26/160 (16.2)		35/506 (6.9)	
Malaria	13/94 (13.8)		70/591 (11.8)	
Pneumonia	9/58 (15.5)		18/242 (7.4)	
URTI	198/1197 (16.5)		20/231 (8.7)	
Gastroenteritis	13/83 (15.7)		1/12 (8.3)	
Undifferentiated fever	25/109 (22.9)		15/217 (6.9)	

^a^‘Other’ clinical diagnosis includes: helminthiasis, ear infections, and non-infectious conditions such as wounds, burns, and malnutrition

CHIKV RT-PCR positivity was rare among asymptomatic children in the community; only 3 out of 435 asymptomatic children sampled during the cross-sectional survey in the same study locations in 2016 were CHIKV RT-PCR positive (0.7, 95% CI 0.22, 2.12). This yielded an overall clinical-to-asymptomatic CHIKV infection ratio of 18:1 during the epidemic year.

### Incidence of CHIKF in the community

We calculated the incidence of CHIKF restricting our analysis to children aged < 16 years whose residence was within the dispensary catchment area. This represented a total of 136,509 person-years of observation (pyo) over the study period at both locations.

The overall CHIKF incidence during the study period was 314 cases/100,000 pyo (95% CI 285, 345; Table 2). An inverse relationship was evident between CHIKF incidence and age, consistent with acquisition of protective immunity against disease due to ongoing CHIKV exposure in this setting (Table 2). CHIKF incidence was 17% higher in Ngerenya than Pingilikani but the difference did not achieve statistical significance (IRR 1.17; 95% CI 0.97, 1.41); furthermore, while excess incidence was observed in Ngerenya during the 2016 CHIKF epidemic, such an increase was not observed in Pingilikani where CHIKF incidence had been gradually declining since 2014 (Table 2). In Pingilikani the highest incidence was observed during the rainy season (April–June) which contrasted with Ngerenya where the highest incidence was during the dry months of January–March (Table 2). We also observed high incidence in populations living closer to the dispensaries which likely reflects access to care (Table 2) as has been previously reported for malaria incidence [33].

**Table 2:** Incidence of CHIKF among children < 16 years in Ngerenya and Pingilikani

	Ngerenya (***N*** = 73,028 pyo)		Pingilikani (***N*** = 63,481 pyo)	
	Incidence (95% CI)	IRRs (95% CI)	Incidence (95% CI)	IRRs (95% CI)
**Overall incidence**	336 (296,381)	–	288 (248,333)	–
**Year**				
2014	–	–	536 (418,676)	Ref
2015	79 (41,138)	Ref	348 (255,464)	0.65 (0.45, 0.94)
2016	920 (774,1086)	11.58 (6.42, 20.89)	274 (192,380)	0.51 (0.34, 0.77)
2017	514 (406,642)	6.47 (3.52, 11.88)	122 (69,198)	0.23 (0.13, 0.39)
2018	127 (72,207)	1.60 (0.76, 3.39)	129 (70,216)	0.24 (0.14, 0.43)
**Sex**				
Female	320 (265,384)	Ref	331 (270,401)	Ref
Male	352 (294,418)	1.10 (0.86, 1.41)	245 (194,306)	0.74 (0.55, 0.99)
**Age (years)**				
< 1	1363 (1030,1770)	Ref	979 (674,1375)	Ref
1 to < 5	658 (549,783)	0.48 (0.35, 0.66)	509 (406,629)	0.52 (0.35, 0.78)
5 to < 10	221 (166,288)	0.16 (0.11, 0.24)	208 (151,280)	0.21 (0.14, 0.33)
10 to 15	35 (16,67)	0.03 (0.01, 0.05)	93 (58,143)	0.10 (0.06, 0.17)
**Distance from dispensary (Km)**				
< 1	881 (599,1251)	Ref	1037 (677,1519)	Ref
1 to < 2	636 (483,822)	0.72 (0.47, 1.12)	855 (605,1173)	0.82 (0.50, 1.36)
2 to < 3	456 (353,580)	0.52 (0.34, 0.79)	883 (700,1099)	0.85 (0.55, 1.33)
3 to < 4	376 (277,498)	0.43 (0.27, 0.67)	207 (116,343)	0.20 (0.11, 0.38)
4 to < 5	181 (120,261)	0.21 (0.12, 0.34)	148 (81,249)	0.14 (0.07, 0.27)
5 to < 6	84 (47,139)	0.10 (0.05, 0.18)	32 (15,59)	0.03 (0.02, 0.06)
**Season**				
Jan - Mar	459 (368,566)	Ref	273 (199,365)	Ref
Apr - Jun	350 (271,445)	0.76 (0.55, 1.05)	433 (338,546)	1.59 (1.09, 2.30)
Jul - Sep	276 (206,362)	0.60 (0.43, 0.85)	281 (205,374)	1.03 (0.68, 1.55)
Oct - Dec	249 (179,338)	0.54 (0.37, 0.79)	147 (91,225)	0.54 (0.32, 0.91)

*Ref – comparator group

### Recurrent CHIKF episodes

**Fig. 3:**
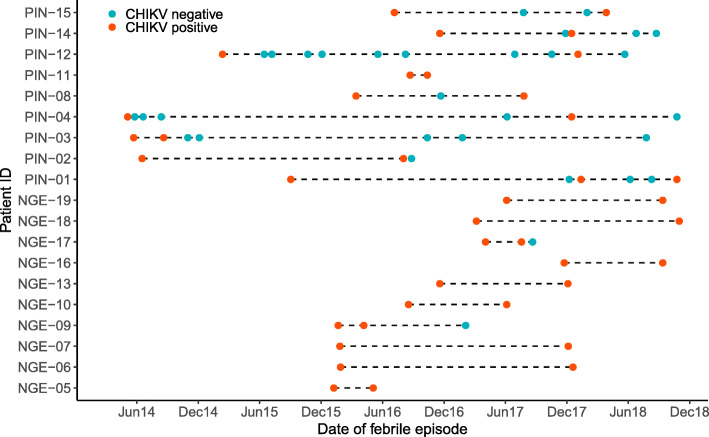
Recurrent CHIKF episodes. The distribution of febrile episodes for each of 19 children who had at least two CHIKV RT-PCR positive febrile episodes is shown. The timing of the CHIKV RT-PCR positive results is shown, with the index episode shown as the first data point in the follow up timeline. Children with prefix ‘NGE’ were resident in Ngerenya and those with ‘PIN’ from Pingilikani, respectively

We determined whether recurrent CHIKF episodes occurred in our study population. To do this we identified the 443 children whose index febrile episode was CHIKV positive and screened all their subsequent samples collected during febrile episodes over the period 2014–2018. We defined recurrent episodes as CHIKV RT-PCR positivity during these subsequent febrile dispensary visits (see Methods). Of the 443 children, 170 presented to the dispensary with fever on at least one other occasion during the study period, contributing a total of 320 subsequent febrile dispensary visits with varying durations since the corresponding index CHIKF episode (Fig. 2). Of the 170 children, 19 (11.2, 95% CI 6.86, 16.90) were CHIKV RT-PCR positive on at least one subsequent febrile episode (Figs. 2 and 3). The duration between the index and the subsequent RT-PCR positive febrile episodes ranged from 2 to 43 months (Fig. 3, Table S2). Eighteen of the 19 CHIKV RT-PCR positive cases were detected during or after the 2016 CHIKF epidemic year. Neither age, sex nor geographic location were statistically associated with recurrent episodes of CHIKF (Chi2 test *p* > 0.05 for all) and the RT-PCR Ct values and clinical diagnoses were comparable between the index and recurrent CHIKF episodes (Figure S2 and Table S2).

### Viral phylogeny

**Fig. 4:**
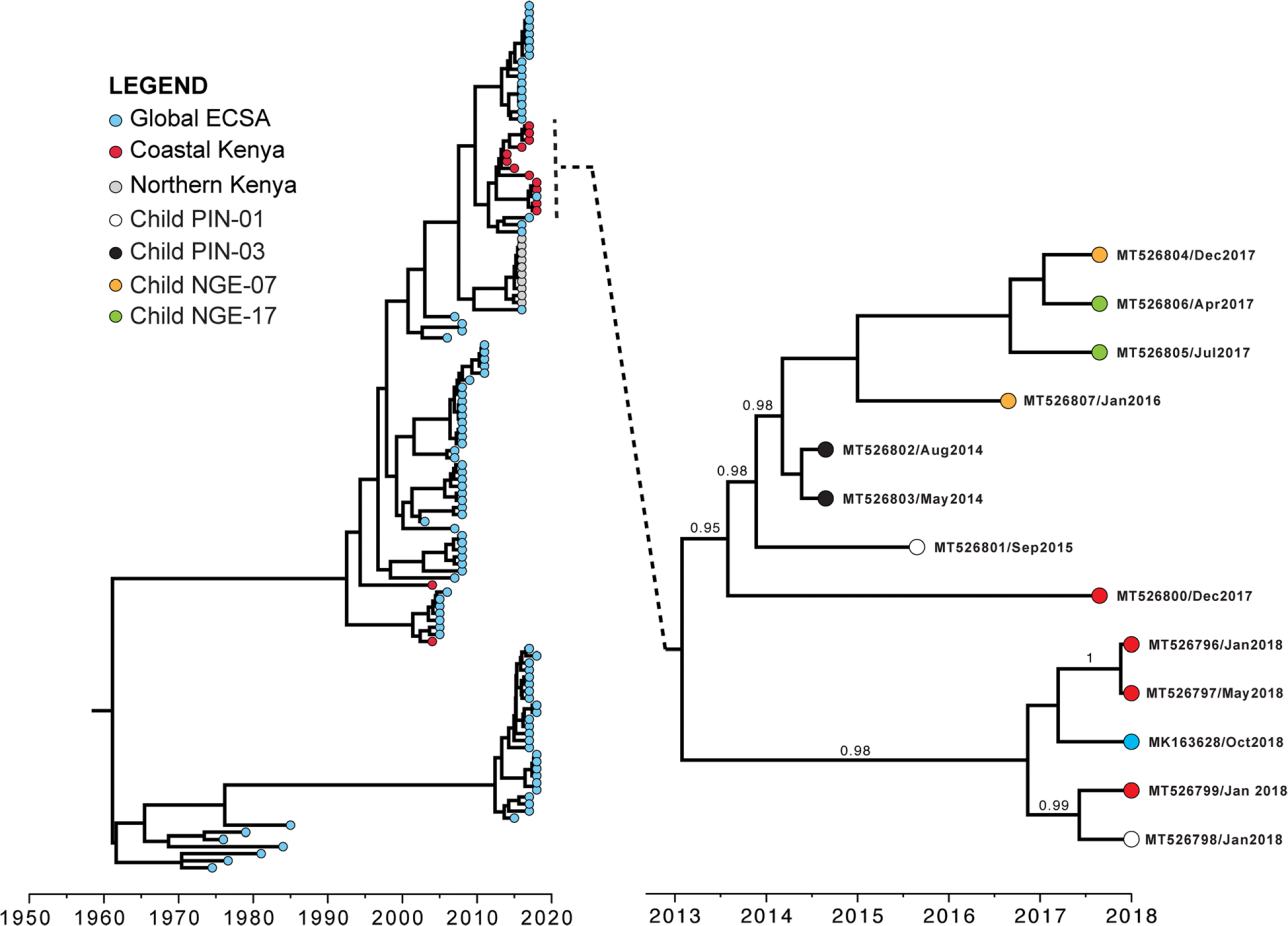
Phylogenetic analysis of CHIKV genomes from Kenya. BEAST MCC tree of 123 ECSA genomes collected across the world, including 24 from Kenya is shown in the left panel. On the right panel is an extraction highlighting the CHIKV sequences generated in this study. The tips are coloured according to geographic location or child identity (as in Fig. 3, for those with paired index-recurrent episode sequence pairs), with posterior probability support shown for select nodes with probability > 0.8. Sample MT526800 was from a child with a single CHIKF episode whose RT-PCR cycle threshold (Ct) value was low (Ct = 19). The same and more detailed MCC tree can be found in Figure S3 in Supplementary Material

We attempted to sequence all index and recurrent CHIKF episode samples from the 19 children (Fig. 3). Complete and partial genome coding sequences were generated from 9 samples, which all mapped to the ECSA genotype, albeit distinct from the clade containing the IOL strains that emerged during the 2004 epidemic and the clade including genomes from CHIKF patients sampled in Mandera, northern Kenya, during the 2016 epidemic (Fig. 4).

The E1 A226V mutation, associated with adaptation and increased transmissibility by *Aedes albopictus* [15]*,* was absent from all sequences sampled in Kenya [23]. Time-resolved phylogenies showed strong temporal clustering of CHIKV sequences from coastal Kenya that may suggest antigenic changes over time driven by acquisition of herd immunity (Fig. 4). Index-recurrent CHIKF episode genome sequence pairs were available for four children with respective time intervals of 2.9, 3.5, 22.3 and 28.3 months between the index and recurrent episodes (Fig. 4). The degree of relatedness of these index-recurrent episode sequence pairs was also time-dependent, with the sequence pair with the longest intervening time interval (28.3 months) being most divergent (Fig. 4). The relatedness of CHIKV genomes from index-recurrent sequence pairs in the same child was similar to the relatedness of CHIKV genomes from different children (Fig. 4), suggesting that these were reinfections rather than relapses.

## Discussion

In summary, we find that CHIKF is endemic in coastal Kenya with the case burden being particularly high in young children aged < 1 year. Our population-based cohort approach in primary healthcare facilities could detect an increase in CHIKF cases during the 2016 epidemic, supporting the utility of such a surveillance framework in the early detection of CHIKF epidemics. We observed a substantial CHIKF incidence during the epidemic and non-epidemic years. However, a significant increase in CHIKF incidence over time was noted in Ngerenya while in Pingilikani (located approximately 40 km south of Ngerenya) incidence had been declining. CHIKF incidence was highest during the rainy season in Pingilikani and during the dry season in Ngerenya suggesting potential differences in the ecology of CHIKF in these locations as has been observed for malaria [27]. *Aedes spp* mosquito vectors for CHIKV are present in Kilifi but their spatial and temporal (by season) distribution in this setting and how this influences risk of CHIKV infection remains to be determined [34].

None of the patients in this study had a clinical diagnosis of CHIKF, and in no instance was arthritis or arthralgia reported in the clinical assessment. This may reflect the rarity of CHIKF-associated joint involvement in children and is consistent with other studies in East Africa [19, 35]. The observed inverse relationship between age and CHIKF incidence suggests that there is an acquisition of protective immunity. However, nineteen children had recurrent CHIKF episodes with viral genome sequences from these recurrent episodes not showing high degrees of relatedness to the index episodes. The relatedness of virus genomes from index and recurrent infections in the same child was similar to the relatedness of virus genomes from different children, suggesting that these were reinfections rather than relapses.

It is plausible that CHIKV infections in children generate immune responses with variable efficacy against clinical disease that may underlie the recurrent CHIKF episodes observed in our setting. Neutralising antibodies that develop following CHIKV infection have been shown to correlate with decreased risk of clinical illness in a longitudinal study in the Philippines [36, 37], though CHIKF cases in that study were all due to the Asian CHIKV genotype. To date, all the CHIKV genomes identified in East Africa (including this study) have been of the ECSA genotype [12, 23], which tend to be more diverse [12] and associated with a higher clinical disease burden than the Asian genotype [38]. We are aware of no longitudinal studies investigating the efficacy of naturally acquired immune responses against CHIKF in East Africa. Only finger-prick whole blood samples for RT-PCR analysis were available for this study, thus precluding any immunological work. Future longitudinal studies with serial blood sampling for immunological assays will help determine the natural course of infection, including duration of viraemia, and identify host and viral factors that may underlie the occurrence of recurrent CHIKF episodes in coastal Kenya.

In contrast to the 2016 CHIKF epidemic viruses from Mandera (northern Kenya) that formed a single well-defined clade, sequences from coastal Kenya were highly diverse and fell within multiple clades whose divergence increased over time. Together, these data suggest ongoing endemic CHIKV transmission at the Kenyan coast which may be sustained through genetically diverse CHIKV strains from local sylvatic transmission cycles [6].

## Conclusions

Our data and that from others in the region [35] further reinforce the need for inclusion of CHIKF high on the list of differential diagnoses to consider when faced with a febrile child at primary healthcare facilities in coastal Kenya. Access to rapid diagnostics would be of value in antibiotic stewardship, and control of transmission in the community would substantially reduce the burden on healthcare facilities. Further studies on the associated clinical outcomes, including the incidence of severe (‘atypical’) manifestations of CHIKF, should help determine the wider public health significance of CHIKF in coastal Kenya.

## Supplementary Information

**Additional file 1.**

## Data Availability

The replication data and analysis scripts for this manuscript shall be made available at the Harvard Dataverse: (https://dataverse.harvard.edu/dataverse/kwtrp). Some of the clinical dataset contain potentially identifying information on participants and is stored under restricted access. Requests for access to the restricted dataset should be made to the Data Governance Committee (dgc@kemri-wellcome.org) of the KEMRI-Wellcome Trust Research Programme.

## Abbreviations

CHIKF: Chikungunya fever
CHIKV: Chikungunya virus
RT-PCR: Reverse transcriptase polymerase chain reaction
ECSA: East, Central and Southern Africa
KHDSS: Kilifi Health and Demographic Surveillance System
Pyo: Person-years observed

## References

[1] Robinson MC (1955). An epidemic of virus disease in Southern Province, Tanganyika territory, in 1952-53. I. Clinical features. Trans R Soc Trop Med Hyg.

[2] Lumsden WH (1955). An epidemic of virus disease in Southern Province, Tanganyika territory, in 1952-53. II. General description and epidemiology. Trans R Soc Trop Med Hyg.

[3] Tritsch SR, Encinales L, Pacheco N, Cadena A, Cure C, McMahon E, et al. Chronic Joint Pain 3 Years after Chikungunya Virus Infection Largely Characterized by Relapsing-remitting Symptoms. J Rheumatol. 2020;47(8):1267–4. 10.3899/jrheum.190162 PMC7938419 31263071

[4] Ross RW (1956). The Newala epidemic. III. The virus: isolation, pathogenic properties and relationship to the epidemic. J Hyg (Lond).

[5] Althouse BM, Guerbois M, Cummings DAT, Diop OM, Faye O, Faye A, Diallo D, Sadio BD, Sow A, Faye O (2018). Role of monkeys in the sylvatic cycle of chikungunya virus in Senegal. Nat Commun.

[6] Eastwood G, Sang RC, Guerbois M, Taracha ELN, Weaver SC (2017). Enzootic circulation of Chikungunya virus in East Africa: serological evidence in non-human Kenyan primates. Am J Trop Med Hyg.

[7] Weaver SC, Lecuit M (2015). Chikungunya virus and the global spread of a mosquito-borne disease. N Engl J Med.

[8] Wahid B, Ali A, Rafique S, Idrees M (2017). Global expansion of chikungunya virus: mapping the 64-year history. Int J Infect Dis.

[9] Voss JE, Vaney MC, Duquerroy S, Vonrhein C, Girard-Blanc C, Crublet E, Thompson A, Bricogne G, Rey FA (2010). Glycoprotein organization of Chikungunya virus particles revealed by X-ray crystallography. Nature.

[10] Rezza G, Weaver SC (2019). Chikungunya as a paradigm for emerging viral diseases: evaluating disease impact and hurdles to vaccine development. PLoS Negl Trop Dis.

[11] Gouglas D, Thanh Le T, Henderson K, Kaloudis A, Danielsen T, Hammersland NC, Robinson JM, Heaton PM, Rottingen JA (2018). Estimating the cost of vaccine development against epidemic infectious diseases: a cost minimisation study. Lancet Glob Health.

[12] Volk SM, Chen R, Tsetsarkin KA, Adams AP, Garcia TI, Sall AA, Nasar F, Schuh AJ, Holmes EC, Higgs S (2010). Genome-scale phylogenetic analyses of chikungunya virus reveal independent emergences of recent epidemics and various evolutionary rates. J Virol.

[13] Chretien JP, Anyamba A, Bedno SA, Breiman RF, Sang R, Sergon K, Powers AM, Onyango CO, Small J, Tucker CJ (2007). Drought-associated chikungunya emergence along coastal East Africa. Am J Trop Med Hyg.

[14] Sergon K, Njuguna C, Kalani R, Ofula V, Onyango C, Konongoi LS, Bedno S, Burke H, Dumilla AM, Konde J (2008). Seroprevalence of Chikungunya virus (CHIKV) infection on Lamu Island, Kenya, October 2004. Am J Trop Med Hyg.

[15] Tsetsarkin KA, Vanlandingham DL, McGee CE, Higgs S (2007). A single mutation in chikungunya virus affects vector specificity and epidemic potential. PLoS Pathog.

[16] Weaver SC, Forrester NL (2015). Chikungunya: evolutionary history and recent epidemic spread. Antivir Res.

[17] Nsoesie EO, Kraemer MU, Golding N, Pigott DM, Brady OJ, Moyes CL, et al. Global distribution and environmental suitability for chikungunya virus, 1952 to 2015. Euro Surveill. 2016;21(20). 10.2807/1560-7917.ES.2016.21.20.30234. 10.2807/1560-7917.ES.2016.21.20.30234 PMC4902126 27239817

[18] Kajeguka DC, Kaaya RD, Mwakalinga S, Ndossi R, Ndaro A, Chilongola JO, Mosha FW, Schioler KL, Kavishe RA, Alifrangis M (2016). Prevalence of dengue and chikungunya virus infections in North-Eastern Tanzania: a cross sectional study among participants presenting with malaria-like symptoms. BMC Infect Dis.

[19] Chipwaza B, Mugasa JP, Selemani M, Amuri M, Mosha F, Ngatunga SD, Gwakisa PS (2014). Dengue and Chikungunya fever among viral diseases in outpatient febrile children in Kilosa district hospital, Tanzania. PLoS Negl Trop Dis.

[20] Hertz JT, Munishi OM, Ooi EE, Howe S, Lim WY, Chow A, Morrissey AB, Bartlett JA, Onyango JJ, Maro VP (2012). Chikungunya and dengue fever among hospitalized febrile patients in northern Tanzania. Am J Trop Med Hyg.

[21] Weller N, Clowes P, Dobler G, Saathoff E, Kroidl I, Ntinginya NE, Maboko L, Loscher T, Hoelscher M, Heinrich N (2014). Seroprevalence of alphavirus antibodies in a cross-sectional study in southwestern Tanzania suggests endemic circulation of chikungunya. PLoS Negl Trop Dis.

[22] LaBeaud AD, Banda T, Brichard J, Muchiri EM, Mungai PL, Mutuku FM, Borland E, Gildengorin G, Pfeil S, Teng CY (2015). High rates of o'nyong nyong and Chikungunya virus transmission in coastal Kenya. PLoS Negl Trop Dis.

[23] Maljkovic Berry I, Eyase F, Pollett S, Konongoi SL, Joyce MG, Figueroa K, Ofula V, Koka H, Koskei E, Nyunja A (2019). Global outbreaks and origins of a Chikungunya virus variant carrying mutations which may increase fitness for Aedes aegypti: revelations from the 2016 Mandera, Kenya outbreak. Am J Trop Med Hyg.

[24] Scott JA, Bauni E, Moisi JC, Ojal J, Gatakaa H, Nyundo C, Molyneux CS, Kombe F, Tsofa B, Marsh K (2012). Profile: the Kilifi health and demographic surveillance system (KHDSS). Int J Epidemiol.

[25] Snow RW, Kibuchi E, Karuri SW, Sang G, Gitonga CW, Mwandawiro C, Bejon P, Noor AM (2015). Changing malaria prevalence on the Kenyan coast since 1974: climate, Drugs and Vector Control. PLoS One.

[26] Mogeni P, Williams TN, Fegan G, Nyundo C, Bauni E, Mwai K, Omedo I, Njuguna P, Newton CR, Osier F (2016). Age, spatial, and temporal variations in hospital admissions with malaria in Kilifi County, Kenya: a 25-year longitudinal observational study. PLoS Med.

[27] Olotu A, Fegan G, Williams TN, Sasi P, Ogada E, Bauni E, Wambua J, Marsh K, Borrmann S, Bejon P (2010). Defining clinical malaria: the specificity and incidence of endpoints from active and passive surveillance of children in rural Kenya. PLoS One.

[28] Lanciotti RS, Kosoy OL, Laven JJ, Panella AJ, Velez JO, Lambert AJ, Campbell GL (2007). Chikungunya virus in US travelers returning from India, 2006. Emerg Infect Dis.

[29] Pabbaraju K, Wong S, Gill K, Fonseca K, Tipples GA, Tellier R (2016). Simultaneous detection of Zika, Chikungunya and dengue viruses by a multiplex real-time RT-PCR assay. J Clin Virol.

[30] Quick J, Grubaugh ND, Pullan ST, Claro IM, Smith AD, Gangavarapu K, Oliveira G, Robles-Sikisaka R, Rogers TF, Beutler NA (2017). Multiplex PCR method for MinION and Illumina sequencing of Zika and other virus genomes directly from clinical samples. Nat Protoc.

[31] Kozlov AM, Darriba D, Flouri T, Morel B, Stamatakis A (2019). RAxML-NG: a fast, scalable and user-friendly tool for maximum likelihood phylogenetic inference. Bioinformatics.

[32] Ndungu FM, Marsh K, Fegan G, Wambua J, Nyangweso G, Ogada E, Mwangi T, Nyundo C, Macharia A, Uyoga S (2015). Identifying children with excess malaria episodes after adjusting for variation in exposure: identification from a longitudinal study using statistical count models. BMC Med.

[33] Bejon P, Williams TN, Nyundo C, Hay SI, Benz D, Gething PW, Otiende M, Peshu J, Bashraheil M, Greenhouse B (2014). A micro-epidemiological analysis of febrile malaria in coastal Kenya showing hotspots within hotspots. Elife.

[34] Karisa J, Muriu S, Omuoyo D, Karia B, Ngari M, Nyamwaya D, et al. Urban Ecology of Arboviral Mosquito Vectors Along the Kenyan Coast. J Med Entomol. 2021;58(1):428–38. 10.1093/jme/tjaa136 PMC7613328 32623459

[35] Waggoner J, Brichard J, Mutuku F, Ndenga B, Heath CJ, Mohamed-Hadley A, Sahoo MK, Vulule J, Lefterova M, Banaei N (2017). Malaria and Chikungunya Detected Using Molecular Diagnostics Among Febrile Kenyan Children. Open Forum Infect Dis.

[36] Yoon IK, Alera MT, Lago CB, Tac-An IA, Villa D, Fernandez S, Thaisomboonsuk B, Klungthong C, Levy JW, Velasco JM (2015). High rate of subclinical chikungunya virus infection and association of neutralizing antibody with protection in a prospective cohort in the Philippines. PLoS Negl Trop Dis.

[37] Yoon IK, Srikiatkhachorn A, Alera MT, Fernandez S, Cummings DAT, Salje H (2020). Pre-existing chikungunya virus neutralizing antibodies correlate with risk of symptomatic infection and subclinical seroconversion in a Philippine cohort. Int J Infect Dis.

[38] Bustos Carrillo F, Collado D, Sanchez N, Ojeda S, Lopez Mercado B, Burger-Calderon R, Gresh L, Gordon A, Balmaseda A, Kuan G (2019). Epidemiological Evidence for Lineage-Specific Differences in the Risk of Inapparent Chikungunya Virus Infection. J Virol.

