# Supplementary material for: Endemic chikungunya fever in Kenyan children: a prospective cohort study
Source: BMC Infect Dis. 2021 Feb 18;21:186. doi: 10.1186/s12879-021-05875-5 (PMC7889702; doi:10.1186/s12879-021-05875-5)
Supplement: Supplementary file 1 — Additional file 1. [file 12879_2021_5875_MOESM1_ESM.docx]

**SUPPLEMENTARY MATERIAL**

**Table of contents**

1. Supplementary methods
2. Table S1: Primers used for CHIKV sequencing
3. Figure S1: Monthly rainfall levels in Kilifi
4. Figure S2: CHIKV RT-PCR cycle threshold values
5. Figure S3: Phylogeny of ECSA genomes included in the analysis
6. Table S2: Details of children with recurrent CHIKF episodes

**Supplementary methods**

Synthesis of cDNA and multiplex tiling PCR

Viral RNA was extracted from clinical samples using the Trizol reagent (Thermo Fischer Scientific) according to manufacturer’s protocol, eluted in 60ul of DNase/RNase free water. RNA was subjected to qRT-qPCR using the QuantiFast RT-PCR +R Kit (Qiagen) for the detection of CHIKV NSP1 gene using the published primers CHIKV 874 (5’-AAAGGGCAAACTCAGCTTCAC-3’), CHIKV 961 (5’-GCCTGGGCTCATCGTTATTC-3’) and CHIKV 899-FAM (5’-*FAM*-CGCTGTGATACAGTGGTTTCGTGTG-*TAMRA*-3’). Each sample was ran in duplicate, 5µl of RNA as template in a 25µl reaction with primers at a final concentration of 400nM and probe 200nM, and cycling conditions of ; RT at 50°C for 20 minutes, activation at 95°C for 15 minutes then 45 cycles of denaturation at 94°C for 15 seconds and annealing at 60°C for 1 minute conditions. The qRT-PCR positive samples were converted to cDNA using SuperScript II First Strand synthesis system for RT-PCR (Invitrogen). Then, a multiplex tiling PCR was conducted using Q5 High Fidelity Hot-Start DNA Polymerase (New England Biolabs) and CHIKV whole genome sequencing primers designed using Primal Scheme (<http://primal.zibraproject.org>; see Supplementary Table 1), prepared in two separate pools as previously described by Quick *et al*; 2017 [1]. Thermocycling conditions included activation at 98 ֯ C for 30 sec, 40 cycles of denaturation at 98 ֯C for 15sec and a combined annealing/ extension at 65 ֯ C for 5 min. Amplicons were ran in 1.5% agarose gels and visualized using the ChemiDoc XRS+ system (Bio-Rad Laboratoties, USA).

Nanopore library preparation

Amplicons from either pool (1 or 2), as described by Quick *et al*; 2017 [1], were purified separately using 1x AMPure XP Beads (Beckman Coulter) and concentrations determined using Qubit dsDNA HS Assay Kit on a Qubit 3.0 fluorimeter (Life Technologies). Pool 1 and 2 amplicons from the same sample were then pooled in a single tube. DNA library preparation was performed on 10 samples and two no-template controls per run using the Ligation Sequencing Kit (SQK-LSK109; Oxford Nanopore Technologies, ONT) and the Native Barcoding Kit (EXP-NBD114, ONT) as per the manufacturer’s recommendation. The library was loaded onto a R9.4 flow cell and sequencing performed for 6 hours on an Mk1B MinION device.

Sequence assembly

Raw signal files encoded in FAST5 format were basecalled using Guppy version 3.4.3 (htttp://nanoporetech.com) to produce FASTQ formatted sequence files. The FASTQ sequences were then demultiplexed and barcodes removed using the Guppy demultiplexer routine. Each demultiplexed set of reads were assigned to its respective sample name. A custom script was used to assemble the consensus sequence as follows. First the reads were aligned to the reference sequence (GenBank accession HQ456255, sampled during the 2004 epidemic in coastal Kenya) using Minimap2 version 2.17-r941. The resulting BAM file was sorted and indexed. BCF-tools version 1.10.2 was used to call variants from the BAM file and to generate a consensus sequence. Reads were aligned to the respective consensus sequence from each sample and the resulting BAM file pileup sorted and indexed. BEDTools was used to calculate the reads coverage and produce a BED file of low coverage regions (less than 10 reads). The raw fast5 files were indexed with Nanopolish version 0.12.2. Nanopolish was then used to polish the consensus sequence and to generate a fresh consensus sequence after the which variants were called and low coverage regions masked using bedtools and based on coverage.

The ONT MinION per-read sequencing error-rate has been characterised by advancement in both sequencing chemistry and base calling methods and the consensus base accuracy now stands at 99.9% or more. Five of the genomes generated using Oxford Nanopore platform in this study (index and recurrent episode samples from NGE07, recurrent episode samples from NGE17, PIN03 and PIN01; see Figure 2 and Table S2) were also sequenced on the Illumina Miseq platform, using the same laboratory workflow. To confirm the agreement in sequencing platforms we mapped the respective MiSeq-generated reads on to ONT Minion consensus generated genomes to confirm the observed mutations. All the MiSeq short reads in each respective sample aligned to the respective genomes with no mismatches indicating inter-sequencing platform congruence.

Phylogenetic analysis

Publicly available CHIKV whole genome sequences were first retrieved from GenBank on 6^th^ April 2020. Sequences without collection dates and location were discarded and the remainder were downloaded and added to the 10 newly sequenced Kilifi genomes (9 from children with recurrent episodes, and 1 from a child with a single CHIKF episode whose RT-PCR cycle threshold (Ct) value was low (Ct=19)). The sequences were subjected to the Chikungunya typing tool (<https://www.genomedetective.com/app/typingtool/chikungunya/>) to determine the genotype, and only sequences belonging to the ECSA genotype (N=123, including the 10 from Kilifi) were retained. The genomes were then aligned using MUSCLE in AliView software (version 1.25) and trimmed to only include the ORFs. Maximum Likelihood phylogenetic trees were generated using RAxML using the GTR substitution model with 4 gamma categories (GTR+G4) [2].

To investigate temporal signal in the ECSA dataset, we regressed root-to-tip genetic distances from this ML tree against sample collection dates using TempEst v1.5.1 ([http://tree.bio.ed.ac.uk](http://tree.bio.ed.ac.uk/)). We estimated time to the most recent common ancestor (tMRCA) and the evolutionary rate of the ECSA genomes using BEAST v1.10.4.  The BEAST analysis  was run for 800 million steps, sampling after every 80000 steps under the following settings; (i) Nucleotide substitution model: GTR+G with no of gamma categories, (ii) base frequencies: empirical, (iii) clock model: uncorrelated relaxed clock with a lognormal distribution and a GMRF Bayesian skyride Tree Prior.

**Table S1: Primers used for CHIKV sequencing**

| Primer Name | Sequence | Pool |
| --- | --- | --- |
| CHKVECSA_1_LEFT | AGACACACGTAGCCTACCAGTT | 1 |
| CHKVECSA_1_RIGHT | ACTGCTTGTAAGTCCCCGATCT | 1 |
| CHKVECSA_2_LEFT | TGTCGGACAGGAAGTACCACTG | 2 |
| CHKVECSA_2_RIGHT | TTCAGTACCTGCTCATCTGCCC | 2 |
| CHKVECSA_3_LEFT | TACTGGGTTGGGTTCGACACAA | 1 |
| CHKVECSA_3_RIGHT | TAAAGGCCTGGGCTCATCGTTA | 1 |
| CHKVECSA_4_LEFT | TCGGTGTTCCATTTAAAGGGCAA | 2 |
| CHKVECSA_4_RIGHT | CTTCCATGTCTTTCCGGCACTC | 2 |
| CHKVECSA_5_LEFT | GCAGAACGCAACGGAATACGAA | 1 |
| CHKVECSA_5_RIGHT | TCTGCTTCTTTTTCTGCGTCCC | 1 |
| CHKVECSA_6_LEFT | AGGACTAGAATCAAATGGTTGTTAAGCA | 2 |
| CHKVECSA_6_RIGHT | GTCTTCACTTGCTCCGCCAAAG | 2 |
| CHKVECSA_7_LEFT | GTTACTGCCCAACCAACAGACC | 1 |
| CHKVECSA_7_RIGHT | TAGTCAAGTCGCCCACCAGTAC | 1 |
| CHKVECSA_8_LEFT | GCAGAGAGGACAGAACACGAGT | 2 |
| CHKVECSA_8_RIGHT | AACGCCTCGTCTACGTACAACA | 2 |
| CHKVECSA_9_LEFT | CGACGTGATGAGACAGAGAGGT | 1 |
| CHKVECSA_9_RIGHT | TTAACACGAGGTCTCCAGGGTC | 1 |
| CHKVECSA_10_LEFT | TACGAAGGCAAAATGCGCACTA | 2 |
| CHKVECSA_10_RIGHT | GCTCCACCTCCCACTCCTTAAT | 2 |
| CHKVECSA_11_LEFT | ACGGAAGGTAAACTGGTATGGAAGA | 1 |
| CHKVECSA_11_RIGHT | CCAGGCCTATTATCCCAGTGGT | 1 |
| CHKVECSA_12_LEFT | TATGTACGCGCATGTATGGGGT | 2 |
| CHKVECSA_12_RIGHT | GCGCTACCCAAGTGACTCTCTT | 2 |
| CHKVECSA_13_LEFT | GGGGAAAGAATGGAATGGCTGG | 1 |
| CHKVECSA_13_RIGHT | CACATGGTGGTTTCAACGCTCT | 1 |
| CHKVECSA_14_LEFT | GGGCGGCTCTCTATTGATCAGA | 2 |
| CHKVECSA_14_RIGHT | ACTGTTTTTGCGGTTCCCACTG | 2 |
| CHKVECSA_15_LEFT | ACGATGAAGAGTGCGTAGTCAAC | 1 |
| CHKVECSA_15_RIGHT | TTCTTTGTCGCGGCAGTAGATG | 1 |
| CHKVECSA_16_LEFT | TACTCAGGAGGGAAAGACAGGC | 2 |
| CHKVECSA_16_RIGHT | TCAATACTTTCCCCCAGGGCAT | 2 |
| CHKVECSA_17_LEFT | CATCAGACGGCTGTGGATATGG | 1 |
| CHKVECSA_17_RIGHT | GTGCAGACTCCTGGGAAGATCT | 1 |
| CHKVECSA_18_LEFT | AGGAGTGCAAAAAGTCAAATGCTC | 2 |
| CHKVECSA_18_RIGHT | TGCCCTAAAGAATCGGACGCTA | 2 |
| CHKVECSA_19_LEFT | GTAATGAGCACCGTACCTGTCG | 1 |
| CHKVECSA_19_RIGHT | AGTCATAACTCGTCGTCCGTGT | 1 |
| CHKVECSA_20_LEFT | TGTCTTCTGAGCTACTAACTTTCGGA | 2 |
| CHKVECSA_20_RIGHT | AGGGACTTTTGGGGTCTCTGAC | 2 |
| CHKVECSA_21_LEFT | CAGGTATCAGTCGCGCAAAGTA | 1 |
| CHKVECSA_21_RIGHT | TTCTGGAATGGGGACGGTACAG | 1 |
| CHKVECSA_22_LEFT | CGAGCGACATTCAATCCGTCAA | 2 |
| CHKVECSA_22_RIGHT | CCATTGGTACTTCCTGTAGTGGC | 2 |
| CHKVECSA_23_LEFT | AGGATAACAACTGAGAATTTAGCAACCT | 1 |
| CHKVECSA_23_RIGHT | GCGCAAGTGAATCATCTTGGCT | 1 |
| CHKVECSA_24_LEFT | ATTTCGATGCCATCATAGCCGC | 2 |
| CHKVECSA_24_RIGHT | ACTTCCATGTTCATCCAAGTGGC | 2 |
| CHKVECSA_25_LEFT | GATCGTCTGACAAAATCCGCGT | 1 |
| CHKVECSA_25_RIGHT | GCAAAGGTGGCCATGGACATTA | 1 |
| CHKVECSA_26_LEFT | AGATGGCAACGAACAGGGCTAA | 2 |
| CHKVECSA_26_RIGHT | CTGAGATCAGCTGGGCAAGTTG | 2 |
| CHKVECSA_27_LEFT | AACTTTTTACAATAGGAGGTACCAGCC | 1 |
| CHKVECSA_27_RIGHT | ATCGATGGTCCCCTTTACGTGT | 1 |
| CHKVECSA_28_LEFT | GTATTTTCGAAGTCAAGCACGAAGG | 2 |
| CHKVECSA_28_RIGHT | TACGGGCTCCTTCATTAGCTCC | 2 |
| CHKVECSA_29_LEFT | ATCCCTACAGGTGCTGGCAAAC | 1 |
| CHKVECSA_29_RIGHT | TCGGGACAGTGAGCTAAGTATGG | 1 |
| CHKVECSA_30_LEFT | GCATCCTTAACATGTTCTCCCCA | 2 |
| CHKVECSA_30_RIGHT | AGTGAATCCCACCGTCAGAGTT | 2 |
| CHKVECSA_31_LEFT | GGGCGGGGCTATTTGTAAGAAC | 1 |
| CHKVECSA_31_RIGHT | TTGAGCCACCGCAATTACACTT | 1 |
| CHKVECSA_32_LEFT | CACCCCTGATCGCACATTAATGT | 2 |
| CHKVECSA_32_RIGHT | GGAGTGTTGGGTGGTCAGGATA | 2 |
| CHKVECSA_33_LEFT | CGCTGGCAAATGTAACATGCAG | 1 |
| CHKVECSA_33_RIGHT | CATACCCACCATCGACAGGAGT | 1 |
| CHKVECSA_34_LEFT | ATGGCCACCCGCATGAGATAAT | 2 |
| CHKVECSA_34_RIGHT | CGGGATCACTGTTACGTGTTCG | 2 |
| CHKVECSA_35_LEFT | TGAGACTCTTACCATGCTGCTGT | 1 |
| CHKVECSA_35_RIGHT | GCATGATTCGGACTTCTCCACG | 1 |
| CHKVECSA_36_LEFT | TCACCGGCGTCTACCCATTTAT | 2 |
| CHKVECSA_36_RIGHT | TCGCCAAATTGTCCTGGTCTTC | 2 |
| CHKVECSA_37_LEFT | CAATGTCTTCAGCCTGGACACC | 1 |
| CHKVECSA_37_RIGHT | GACATGTCCGTTAAAGAGGGCG | 1 |
| CHKVECSA_38_LEFT | GCTGCCAAATAGCAACAAACCC | 2 |
| CHKVECSA_38_RIGHT | GCCGGGTAGTTGACTATGTGGT | 2 |
| CHKVECSA_39_LEFT | TGCAAATCTCTTTCTCGACGGC | 1 |
| CHKVECSA_39_RIGHT | TACAGTGTGTCTCTTAGGGGACA | 1 |
| CHKVECSA_40_LEFT | CCCTAAGAGACACACTGTACATAGC | 2 |
| CHKVECSA_40_RIGHT | TCGTGGAAGAGTTCGGTATGCT | 2 |
| CHKVECSA_41_LEFT | AACAGAAAACCATAAACAGAAGTAGTTCAA | 1 |
| CHKVECSA_41_RIGHT | AAAACAAAATAACATCTCCTACGTCCCT | 1 |

**Figure S1: Monthly rainfall levels in Kilifi.** Presented are the monthly rainfall levels in Kilifi county between 2014 and 2018. Data are from a weather station in the environs of Kilifi town.

**Figure S2: CHIKV RT-PCR cycle threshold values.** The plots compare the mean (horizontal line) range of RT-PCR cycle threshold (Ct) values from all 443 CHIKF cases by dispensary location (A), or with respect to the episode type i.e. index or recurrent. No statistically significant differences were observed for both these comparisons.

**Figure S3: Phylogeny of ECSA genomes included in the analysis.** Maximum clade credibility (MCC) tree inferred from 123 ECSA genomes collected across the world, including 24 from Kenya are shown.

**Table S2: Details of children with recurrent CHIKF episodes**

| Location | Patient ID | Sample ID | Date of visit | RT-PCR Ct value | Months between episodes | Clinical diagnosis |
| --- | --- | --- | --- | --- | --- | --- |
| Ngerenya | NGE17 | 4908* | 03-Apr-17 | 37.4 | Index | URTI |
|  |  | 5188* | 19-Jul-17 | 37.2 | 3.5 | URTI |
| Ngerenya | NGE05 | 3083 | 08-Jan-16 | 37.2 | Index | Malaria |
|  |  | 3595 | 04-May-16 | 36.6 | 3.8 | URTI |
| Ngerenya | NGE06 | 3207 | 28-Jan-16 | 34.4 | Index | URTI |
|  |  | 5634 | 19-Dec-17 | 35.9 | 22.7 | URTI |
| Ngerenya | NGE07 | 3200* | 26-Jan-16 | 36.1 | Index | URTI |
|  |  | 5514* | 05-Dec-17 | 36.0 | 22.3 | Gastroenteritis |
| Ngerenya | NGE09 | 3170 | 21-Jan-16 | 36.0 | Index | Undifferentiated fever |
|  |  | 3495 | 06-Apr-16 | 37.6 | 2.5 | Undifferentiated fever |
| Ngerenya | NGE10 | 4076 | 17-Aug-16 | 34.0 | Index | Gastroenteritis |
|  |  | 5078 | 05-Jun-17 | 35.8 | 9.6 | URTI |
| Ngerenya | NGE13 | 4453 | 18-Nov-16 | 36.5 | Index | URTI |
|  |  | 5491 | 04-Dec-17 | 36.0 | 12.5 | URTI |
| Ngerenya | NGE16 | 5409 | 23-Nov-17 | 35.7 | Index | Gastroenteritis |
|  |  | 6557 | 12-Sep-18 | 34.7 | 9.6 | URTI |
| Ngerenya | NGE18 | 4765 | 07-Mar-17 | 37.1 | Index | URTI |
|  |  | 6740 | 31-Oct-18 | 35.4 | 19.8 | URTI |
| Ngerenya | NGE19 | 5065 | 05-Jun-17 | 36.3 | Index | URTI |
|  |  | 6559 | 12-Sep-18 | 36.3 | 15.2 | URTI |
| Pingilikani | PIN01 | 75443* | 02-Sep-15 | 35.5 | Index | Undifferentiated fever |
|  |  | 84807* | 12-Jan-18 | 19.0 | 28.3 | Undifferentiated fever |
|  |  | 89314 | 24-Oct-18 | 34.7 | 37.6 | Malaria |
| Pingilikani | PIN02 | 67660 | 17-Jun-14 | 36.0 | Index | Malaria |
|  |  | 79545 | 02-Aug-16 | 37.5 | 25.5 | Malaria |
| Pingilikani | PIN04 | 66857 | 05-May-14 | 31.5 | Index | Undifferentiated fever |
|  |  | 84601 | 15-Dec-17 | 38.2 | 43.3 | Malaria |
| Pingilikani | PIN12 | 72066 | 11-Feb-15 | 36.3 | Index | Malaria |
|  |  | 84717* | 03-Jan-18 | 34.0 | 34.7 | Malaria |
| Pingilikani | PIN15 | 79039 | 06-Jul-16 | 36.8 | Index | Malaria |
|  |  | 85705 | 28-Mar-18 | 36.9 | 20.7 | Malaria & URTI |
| Pingilikani | PIN08 | 77656 | 14-Mar-16 | 37.0 | Index | Undifferentiated fever |
|  |  | 83382 | 26-Jul-17 | 37.1 | 16.4 | Malaria |
| Pingilikani | PIN03 | 67114* | 23-May-14 | 35.4 | Index | Malaria |
|  |  | 69219* | 20-Aug-14 | 37.7 | 2.9 | Malaria & URTI |
| Pingilikani | PIN11 | 79880 | 22-Aug-16 | 35.8 | Index | Undifferentiated fever |
|  |  | 80541 | 12-Oct-16 | 34.6 | 1.7 | URTI |
| Pingilikani | PIN14 | 80879 | 18-Nov-16 | 36.9 | Index | Pneumonia |
|  |  | 84599 | 15-Dec-17 | 37.8 | 12.9 | Pneumonia |

*****Samples for which genome sequences were successfully obtained.

**References**

1. Quick J, Grubaugh ND, Pullan ST, Claro IM, Smith AD, Gangavarapu K, Oliveira G, Robles-Sikisaka R, Rogers TF, Beutler NA *et al*: **Multiplex PCR method for MinION and Illumina sequencing of Zika and other virus genomes directly from clinical samples**. *Nat Protoc* 2017, **12**(6):1261-1276.

2. Kozlov AM, Darriba D, Flouri T, Morel B, Stamatakis A: **RAxML-NG: a fast, scalable and user-friendly tool for maximum likelihood phylogenetic inference**. *Bioinformatics* 2019, **35**(21):4453-4455.
